# Neutrophils Are Resistant to *Yersinia* YopJ/P-Induced Apoptosis and Are Protected from ROS-Mediated Cell Death by the Type III Secretion System

**DOI:** 10.1371/journal.pone.0009279

**Published:** 2010-02-18

**Authors:** Justin L. Spinner, Keun Seok Seo, Jason L. O'Loughlin, Jennifer A. Cundiff, Scott A. Minnich, Gregory A. Bohach, Scott D. Kobayashi

**Affiliations:** Department of Microbiology, Molecular Biology and Biochemistry, University of Idaho, Moscow, Idaho, United States of America; University of California Merced, United States of America

## Abstract

**Background:**

The human innate immune system relies on the coordinated activity of macrophages and polymorphonuclear leukocytes (neutrophils or PMNs) for defense against bacterial pathogens. *Yersinia* spp. subvert the innate immune response to cause disease in humans. In particular, the *Yersinia* outer protein YopJ (*Y. pestis* and *Y. pseudotuberculosis*) and YopP (*Y. enterocolitica*) rapidly induce apoptosis in murine macrophages and dendritic cells. However, the effects of *Yersinia* Yop J/P on neutrophil fate are not clearly defined.

**Methodology/Principal Findings:**

In this study, we utilized wild-type and mutant strains of *Yersinia* to test the contribution of YopJ and YopP on induction of apoptosis in human monocyte-derived macrophages (HMDM) and neutrophils. Whereas YopJ and YopP similarly induced apoptosis in HMDMs, interaction of human neutrophils with virulence plasmid-containing *Yersinia* did not result in PMN caspase activation, release of LDH, or loss of membrane integrity greater than PMN controls. In contrast, interaction of human PMNs with the virulence plasmid-deficient *Y. pestis* strain KIM6 resulted in increased surface exposure of phosphatidylserine (PS) and cell death. PMN reactive oxygen species (ROS) production was inhibited in a virulence plasmid-dependent but YopJ/YopP-independent manner. Following phagocytic interaction with *Y. pestis* strain KIM6, inhibition of PMN ROS production with diphenyleneiodonium chloride resulted in a reduction of PMN cell death similar to that induced by the virulence plasmid-containing strain *Y. pestis* KIM5.

**Conclusions:**

Our findings showed that *Yersinia* YopJ and/or YopP did not induce pronounced apoptosis in human neutrophils. Furthermore, robust PMN ROS production in response to virulence plasmid-deficient *Yersinia* was associated with increased PMN cell death, suggesting that *Yersinia* inhibition of PMN ROS production plays a role in evasion of the human innate immune response in part by limiting PMN apoptosis.

## Introduction

The cellular arm of the human innate immune system includes the myeloid progenitor-derived mononuclear and polymorphonuclear phagocytes, which recognize and eliminate a diverse array of bacterial pathogens. The mononuclear phagocytes (monocytes, macrophages and dendritic cells) produce proinflammatory cytokines and are capable of presenting antigen to CD4+ T cells via class II MHC [1]. Polymorphonuclear leukocytes (neutrophils or PMNs) are the most abundant circulating leukocyte and essential for removal of pathogens [2]. PMNs are recruited to sites of infection and rapidly phagocytose invading microbes. Following phagocytosis, PMNs destroy pathogens by releasing highly microbicidal NADPH oxidase-derived reactive oxygen species (ROS) and a broad array of potent antimicrobial components into the pathogen-containing vacuoles [2]. Uptake of bacteria induces a form of PMN cell death termed phagocytosis-induced cell death (PICD) that is dependent upon ROS production and is important for resolution of infection and inflammation [3]–[8]. Macrophage clearance of apoptotic PMNs prevents release of cytotoxic granule components and limits subsequent damage to surrounding healthy tissues [2], [4], [6]–[8]. Overall, the innate immune system is highly efficient and essential for human health and subversion of the innate immune system is linked to increased bacterial pathogenicity [9].

The genus *Yersinia* contains three species pathogenic for humans and rodents (*Y. enterocolitica*, *Y. pseudotuberculosis*, and *Y. pestis*). *Y. enterocolitica* and *Y. pseudotuberculosis* are transmitted by fecal-oral route and cause gastrointestinal syndromes, lymphadenitis, and septicemia [10]. *Y. pestis* is typically transmitted through the bite of an infected flea and causes bubonic and/or septicemic plague [11]. Alternatively, a rare but highly lethal pneumonic form of plague may result following inhalation of aerosolized droplets containing *Y. pestis* [11]. Despite epidemiologic differences, *Yersinia* pathogenesis is dependent upon the presence of a homologous virulence plasmid termed pCD1 in *Y. pestis* and pYV in *Y. enterocolitica* and *Y. pseudotuberculosis*. The *Yersinia* virulence plasmid encodes a type III secretion system (TTSS) and 6 known effector proteins termed *Yersinia* outer proteins (Yops) [11]. *In vivo*, *Y. pestis* injects Yops into host immune cells with a preference for dendritic cells, macrophages, and PMNs [12]. Once within the cell, the Yop proteins cooperatively interfere with host cell signal transduction pathways [13]. In particular, the effector protein YopJ (YopP in *Y. enterocolitica*) has been shown to be an acetyltransferase [14]–[16], a deubiquitinating cysteine protease [17], [18], and required for cleavage of the caspase-8 substrate BID [19]. *Yersinia* YopJ and YopP have been shown to rapidly activate programmed cell death (apoptosis) in macrophages and dendritic cells, but not human epithelial cells [19]–[27]. Sequence polymorphisms in the N-terminus of the YopJ/YopP proteins result in higher host cell translocation efficiency of YopP compared to YopJ [28], [29]. The increased delivery of YopP results in increased J774A.1 macrophage and dendritic cell death [28], [29]. Increased host cell cytotoxicity attenuates *Yersinia* infection in mice [28], [30], indicating that a controlled level of immune cell apoptosis is required for *Yersinia* virulence. However, *Y. pestis* induction of apoptosis in human PMNs and human monocyte-derived macrophages (HMDMs) has not been investigated directly. Thus, we evaluated human PMN and HMDM cell fate following incubation with *Yersinia* expressing YopJ or YopP. Our findings show that YopP and (to a lesser extent) YopJ are cytotoxic to HMDMs but not human PMNs. Furthermore, *Yersinia* TTSS-dependent inhibition of human PMN ROS production is associated with reduced PMN apoptosis. Reduction of PMN apoptosis potentially delays the acute inflammatory response and facilitates *Yersinia* persistence via intracellular and extracellular survival [31], [32] at the site of infection.

## Materials and Methods

### Ethics Statement

Human monocytes and PMNs were isolated from heparinized venous blood of healthy donors in accordance with a protocol approved by the University of Idaho Institutional Review Board for Human Subjects (approval number 05-056). Donors were informed of the procedure risks and provided written consent prior to enrollment.

### Macrophage and PMN Cell Isolation and Culture

The murine macrophage-like cell line J774A.1 cells were cultured at 37°C under 5% CO_2_ in Dulbecco's Modified Eagle Medium (DMEM; Invitrogen, Carlsbad, CA) supplemented with 10% heat-inactivated fetal bovine serum (FBS; Thermo Fisher Scientific, Waltham, MA), 100 U/ml penicillin, 100 µg/mL streptomycin, and 2 mM L-glutamine (Invitrogen). For HMDM isolation, freshly isolated heparinized human blood was underlaid with an equal volume of Ficoll-Paque PLUS (Amersham Biosciences, Piscataway, NJ), and centrifuged at 485×g for 25 min. The buffy coat layer was removed by aspiration and diluted with Dulbecco's Phosphate Buffered Saline (DPBS; Invitrogen). The mixture was centrifuged for 25 min at 216×g and the cell pellet suspended in RPMI medium 1640 (Invitrogen) supplemented with 10% FBS, 100 U/ml penicillin, 100 µg/mL streptomycin, and 2 mM L-glutamine (Invitrogen). Cells were allowed to adhere and differentiate for 4–6 days at 37°C under 5% CO_2_ in tissue culture flasks. HMDM purity was determined by staining with CD14 and CD68 antibodies conjugated to phycoerythrin (Invitrogen) and analysis by flow cytometry (FACSCalibur, BD Biosciences, San Jose, CA) as described previously [33]. HMDM preparations routinely contained 90%–97% HMDMs. Human PMN isolation was performed as described previously [32]. PMNs were isolated fresh the day of the assay and seeded into autologous normal human serum (NHS)-coated wells of 96 well tissue culture plates. J774A.1 cells and HMDMs were washed, enumerated, and seeded into wells of 96 well tissue culture plates ∼15 hours prior to the start of each experiment in antibiotic free RPMI 1640 supplemented with 10% FBS. Immediately prior to the assay, J774A.1 cells and HMDMs were washed and medium was replaced with RPMI 1640 supplemented with 2.0% FBS.

### Bacterial Strains and Culture Conditions

Liquid cultures of *Yersinia* were grown at 37°C from viable frozen stocks and routinely verified as previously described [32]. Strains used were: *Y. enterocolitica* 8081v (pYV+), and the *Y. pestis* strains KIM5 (pCD1+), KIM5 YopJ-YopP (pCD1+, *yopJ* replaced with *Y. enterocolitica yopP*), KIM5Δ*yopJ* (pCD1+, Δ*yopJ*), and KIM6 (pCD1-). For assays using J774A.1 cells and HMDMs, bacteria were washed and re-suspended in RPMI 1640 supplemented with 2% FBS. For assays using PMNs, bacteria were opsonized in 5% NHS. For all experiments, opsonized bacteria were added to leukocytes (50∶1 ratio) in 96-well tissue culture plates and centrifuged at 130×g for 3 min to synchronize phagocytosis (represents initial time 0 [T0]). Plates were incubated for 1 h at 37°C under 5% CO_2_ before addition of gentamicin (100 µg/mL) to inhibit extracellular bacterial growth.

### Generation of *Y. pestis* KIM5Δ*yopJ* Deletion Mutant Strain

The KIM5Δ*yopJ* gene deletion strain was created using a modified Red recombinase system as described previously [31], [34]. The YopJ gene deletion cassette was generated by PCR using primers YopJKOF and YopJKOR (Table 1), and pKD4 as the template [34]. The kanamycin resistance gene was removed by the FLP site-specific recombinase encoded on pCP20 (cured by growth at 39°C) as described [34]. The KIM5Δ*yopJ* gene deletion strain was verified by PCR, sequencing (Sequetech, Mountain View, CA), and western blot analysis with a YopJ/YopP affinity-purified polyclonal antibody (rabbit) raised using the conserved peptide sequence CLSDGENPLPHDKLD (GenScript Corporation, Piscataway, NJ). The confirmed DNA sequence was deposited into GenBank (GU066315).

**Table 1:** Oligonucleotides used in this study.

Name	Sequence (5′-3′)
YopJKOF	ATGATCGGACCAATATCACAAATAAATATCTCCGGTGTGTAGGCTGGAGCTGCTTC
YopJKOR	TTATACTTTGAGAAGTGTTTTATATTCAGCTATTCTCATATGAATATCCTCCTTAG
YopPF	AAGCTTGTGCTGCCCGTCTGTTCCGGG
YopPR	CCAGTGCGTGGGACAGGCAGGCAATCCGGAGCG
kanRF	TCCGTGTCGACGTGTAGGCTGGAGCTGCTTC
kanRR	ACGGAGAGCTCCATATGAATATCCTCCTTAG
YopP/J-kanRF	TAGGTATGATAGGAGTTATTGGGAATTTTTGTTCGAGTGCTGCCCGTCTGTTCCGG
YopP/J-kanRR	GATTCCGGCGTAGAACCCCAGTTAATCTGTTTCTTCCATATGAATATCCTCCTTAG

### *Y. pestis* KIM5 YopJ-YopP Open Reading Frame Exchange

Exchange of *Y. pestis* KIM5 YopJ with *Y. enterocolitica* 8081v YopP was by double homologous recombination. The YopP gene was amplified from *Y. enterocolitica* 8081v using primers YopPF and YopPR (Table 1). The amplified YopP gene was cloned into the pGEM-T Easy Vector (Promega, Madison, WI). The kanamycin resistance cassette from pKD4 [34] was amplified using primers kanRF and kanRR (Table 1). The pGEM-T Easy Vector containing YopP and the amplified kanamycin resistance gene were digested with *Sal*I (NEB, Ipswich, MA) and *Sac*I (Fermentas, Glen Burnie, MD) and ligated. Successful ligation created a pGEM-T Easy Vector with the YopP open reading frame immediately upstream of the kanamycin resistance gene. YopP-kanamycin, flanked by 36-nt of homology to the open reading frame of YopJ, was PCR amplified from the modified pGEM-T Easy Vector using primers YopP/J-kanRF and YopP/J-kanRR (Table 1). The YopJ replacement cassette was electroporated into *Y. pestis* KIM5 containing pKD46 [34]. Double homologous recombination was selected for by kanamycin resistance and verified by PCR and DNA sequencing. Plasmid pKD46 was cured from selected colonies and the kanamycin resistance gene was removed by an FLP site-specific recombinase [34]. The final *Y. pestis* KIM5 strain expressing *Y. enterocolitica* YopP in place of native YopJ was verified by PCR and DNA sequencing (GenBank GU066314) using primers upstream and downstream of the YopJ locus, and internal YopP-specific primers. Western blot analysis was performed with a YopJ-YopP affinity-purified polyclonal antibody as described above.

### Cell Cytotoxicity and Membrane Integrity Assays

Cell lysis was quantified by lactose dehydrogenase (LDH) activity using the Cytotoxicity Detection Kit^Plus^ (Roche Applied Science, Indianapolis, IN). Cell membrane integrity was measured using the LIVE/DEAD Viability/Cytotoxicity Kit for mammalian cells (Invitrogen) per manufacturer's instructions. Briefly, 2×10^4^ cells (J774A.1, HMDMs, or PMNs)/assay were seeded into wells prior to addition of bacteria. Cells were allowed to incubate for 3–48 h. LDH activity was measured with a microplate fluorometer (SpectraMax M2; Molecular Devices, Sunnyvale, CA). For membrane integrity, ethidium homodimer-1 (EthD-1; 4.5 µM final concentration) was added at the indicated time points and allowed to incubate for 30 min at room temp. Fluorescence was measured using a microplate fluorometer, excitation and emission of 530 and 645 nm, respectively. The percent LDH release and the percent membrane integrity was calculated using the formula: (experimental release – T0 background)/(maximum release – T0 background) ×100. The T0 background control represents the amount of fluorescence from samples immediately after seeding of cells. The maximum release represents the value from detergent-lysed cells. Experiments were repeated at least three times in triplicate.

### Annexin V Staining of PMNs

PMN phosphatidylserine (PS) externalization at indicated time points was measured with annexin V-FITC (Annexin V-FITC Apoptosis Detection kit II; BD Biosciences, San Jose, CA) as described previously [32].

### Measurement of Caspase Activity

Caspase-3, -8, -9, and -2 activity were measured with ApoAlert caspase profiling plates (Clontech Laboratories, Mountain View, CA) per manufacturer's instructions. Briefly, J774A.1 cells, HMDMs, or PMNs were seeded at 2×10^5^ cells/well prior to addition of bacteria. Incubation was continued for 3, 6, or 24 h. Fluorescence of the lysed cellular supernatants in the ApoAlert caspase profiling plates was read in a microplate fluorometer, by use of excitation and emission wavelengths of 380 and 460 nm, respectively.

### Assay for PMN Reactive Oxygen Species (ROS) Production

PMN ROS production was measured using a published fluorometric method as previously described [32]. Inhibition of PMN ROS production with diphenyleneiodonium chloride (DPI; Sigma-Aldrich, Saint Louis, MO) was performed as described [35].

### Statistics

Statistics were performed with a one-way ANOVA with Tukey's post test using GraphPad Prism, version 5.0 for Windows (GraphPad Software, San Diego, CA). Differences were declared at *P*<0.05.

## Results

### *Yersinia* YopJ/YopP-Dependent J774A.1 and HMDM Cell Death

**Figure 1:**
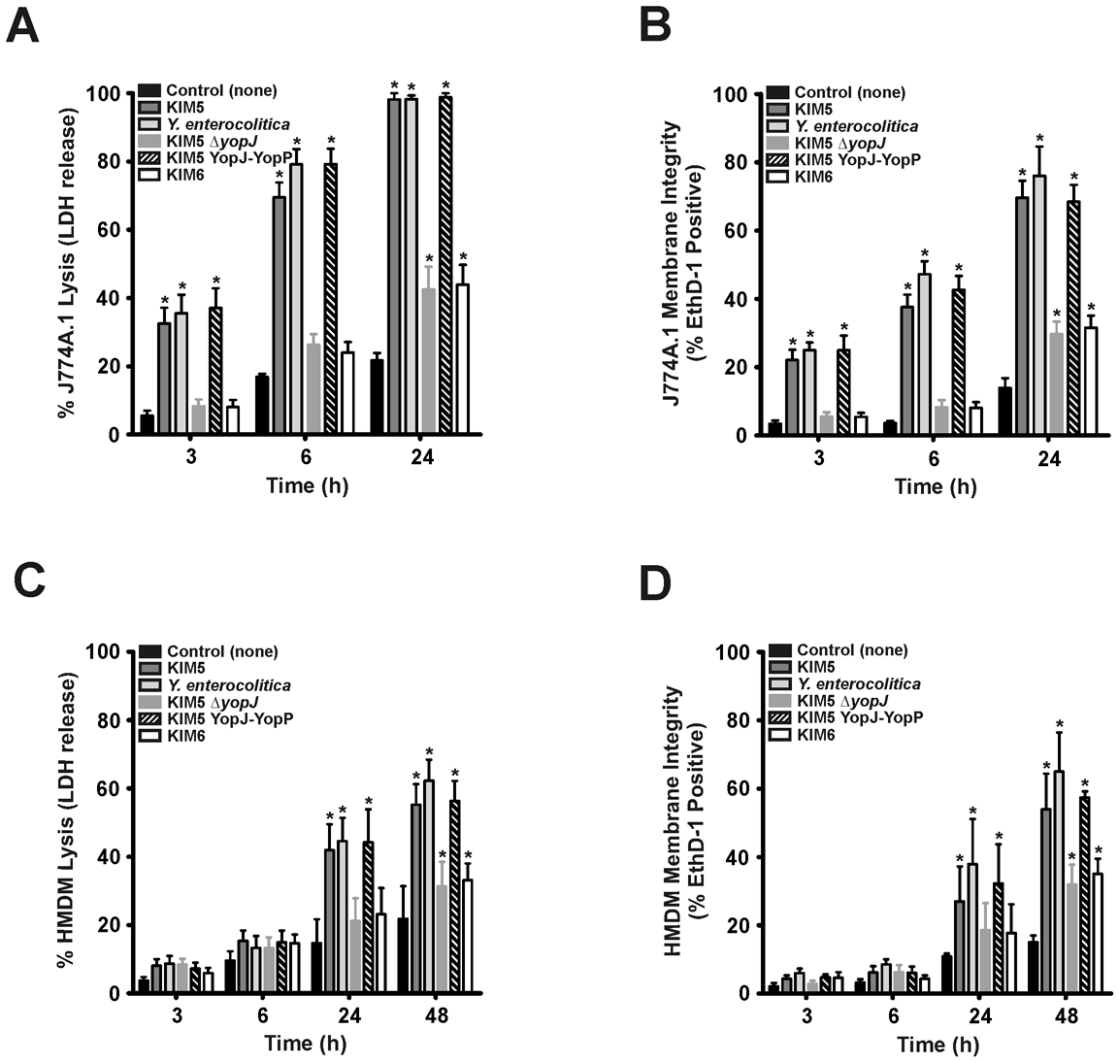
Role of YopJ and YopP in J774A.1 macrophage*-*like and human MDM cell death. *Y. pestis* strains with the virulence plasmid expressing YopJ (KIM5), YopP (KIM5 YopJ-YopP), or lacking YopJ (KIM5Δ*yopJ*), along with *Y. enterocolitica* 8081v and the virulence plasmid–deficient strain (KIM6) were grown at 37°C to induce expression of the TTSS and Yops. *Yersinia* strains were combined with J774A.1 and HMDM cells and compared to detergent lysed controls as described in Materials and Methods. The percentage of J774A.1 cell death following incubation alone or with *Yersinia* strains at the indicated time was determined by (**A**) LDH release into the supernatant and by (**B**) EthD-1 staining of J774A.1 cells. HMDM cell death was determined by measuring (**C**) LDH release and (**D**) EthD-1 uptake into HMDMs. The results are expressed as the mean ± SEM of at least three experiments. *, represents difference from J774A.1 or HMDM controls (*P*<0.05).

To evaluate a role for YopJ and YopP in induction of cell death, we measured LDH release and membrane integrity via EthD-1 staining in the murine J774A.1 cell line and HMDMs following interaction with *Yersinia*. Incubation of J774A.1 cells or HMDMs with KIM5, *Y. enterocolitica*, or KIM5 YopJ-YopP resulted in YopJ- or YopP-dependent LDH release and EthD-1 staining (Fig. 1); however, HMDM cell death was delayed between 24–48 h of incubation compared to J774A.1 cells (Fig. 1c and 1d). In contrast, KIM6 or KIM5Δ*yopJ* incubation with J774A.1 cells or HMDMs resulted in decreased LDH release and EthD-1 staining (Fig. 1, *e.g.*, at 24 h KIM5 and *Y. enterocolitica* induced 98.2±3.2% and 98.2±1.9% of J774A.1 LDH release, respectively, compared to KIM5Δ*yopJ* and KIM6 which induced 42.5±11.4% and 43.9±13.0% of J774A.1 LDH release, respectively).

### *Yersinia* YopJ/YopP Induces Caspase Activation in J774A.1 Macrophages and HMDMs

**Figure 2:**
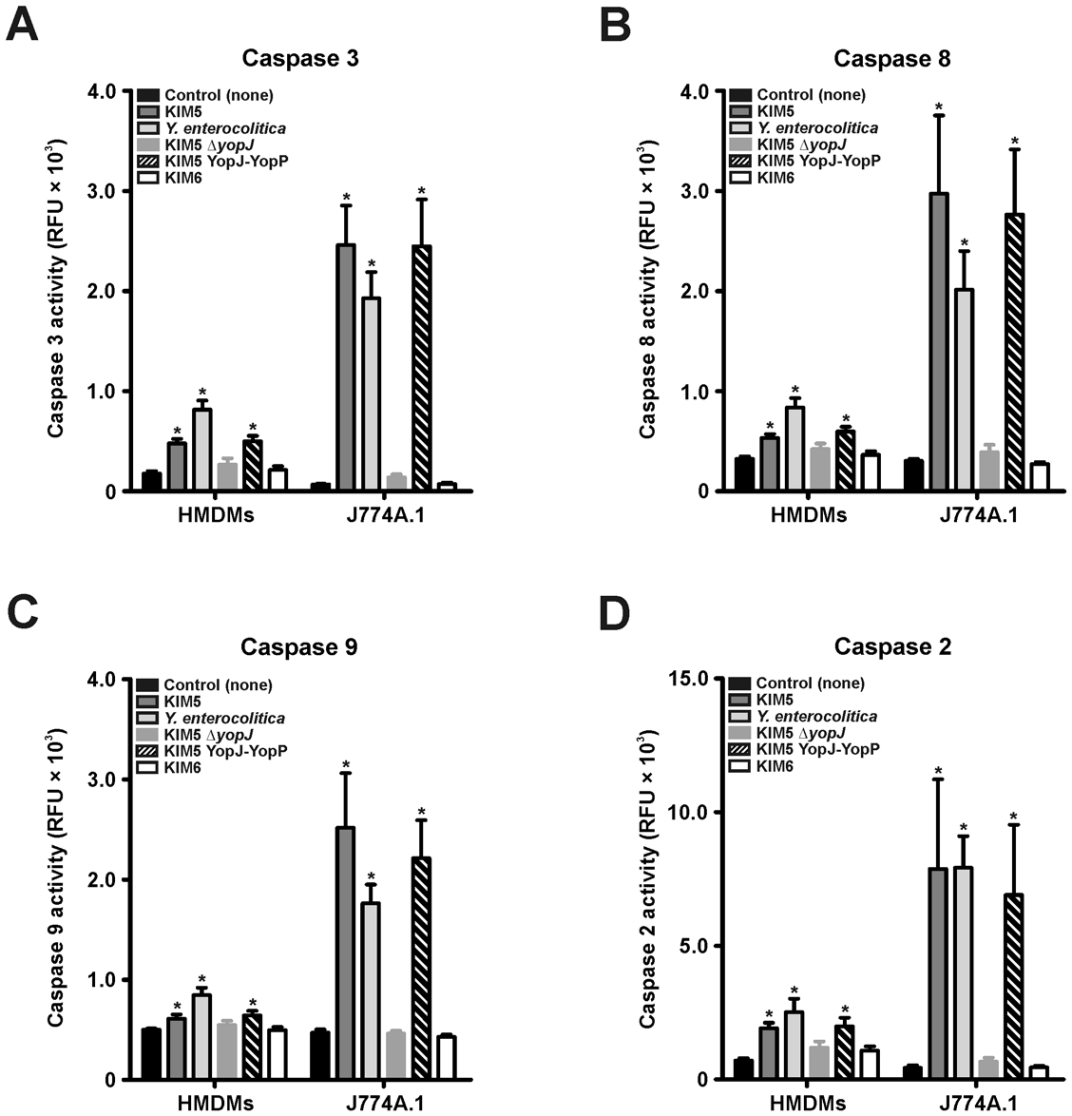
Induction of caspase activity is YopJ/YopP dependent but delayed in human MDMs compared to J774A.1 cells. HMDMs and J774A.1 cells were incubated alone or with *Y. pestis* KIM5, KIM5 YopJ-YopP, KIM5Δ*yopJ*, KIM6 and *Y. enterocolitica* 8081v grown at 37°C. Caspase-3, -8, -9, and -2 activity was measured after 6 h of incubation as described in *Materials and Methods* and is expressed in relative fluorescence units (RFLU). Results are expressed as the mean ± SEM of three experiments. *, represents difference from HMDM and J774A.1 controls (*P*<0.05).

To assess the association of YopJ and YopP with caspase activation in macrophages, we measured caspase-3, -8, -9, and -2 activity following 3 h (data not shown) and 6 h incubation with *Yersinia*. All caspases tested were rapidly activated in J774A.1 macrophages after incubation with KIM5, *Y. enterocolitica*, or KIM5 YopJ-YopP but not KIM6 or KIM5Δ*yopJ* (Fig. 2). HMDMs incubated with *Yersinia* did not result in activation of any caspase tested until ∼6 h (Fig. 2). At 6 h, all caspases tested were activated in HMDMs by YopJ/YopP expressing strains but not by KIM6 or KIM5Δ*yopJ* (Fig. 2). Delayed caspase activation by YopJ/YopP in HMDMs relative to J774A.1 cells corresponds with the observed delays in HMDM lysis and EthD-1 staining (Fig. 1).

### Effects of YopJ/YopP on Neutrophil Viability

**Figure 3:**
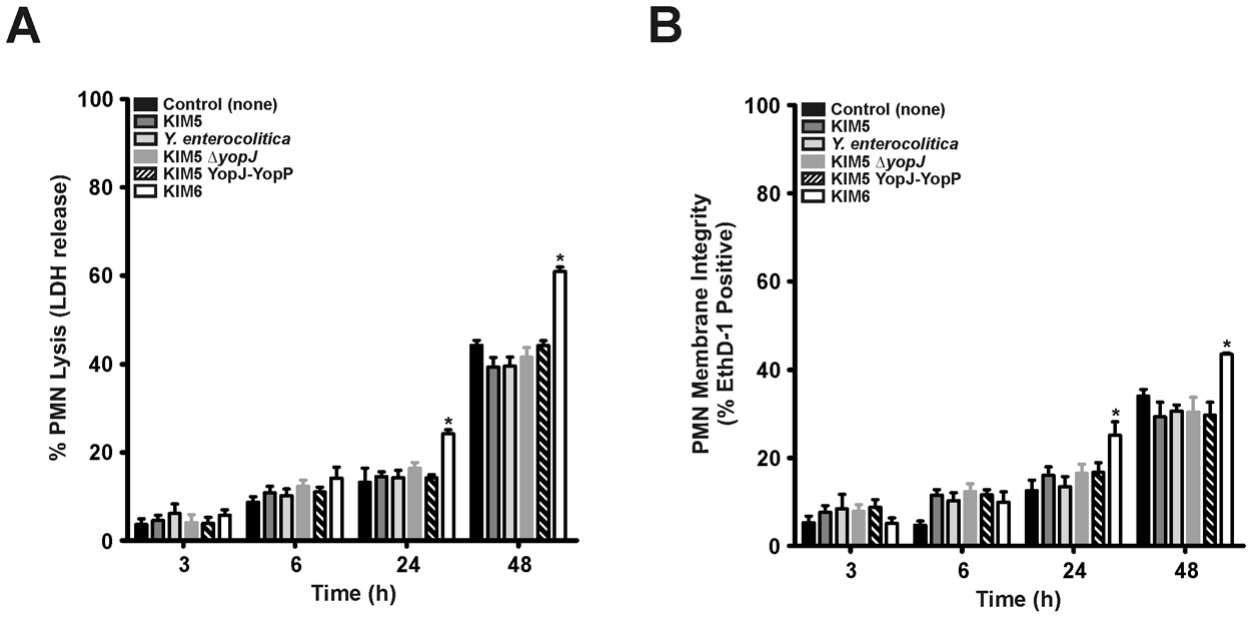
Role of YopJ and YopP in human PMN cell death. PMNs were incubated alone or with *Y. pestis* KIM5, KIM5 YopJ-YopP, KIM5Δ*yopJ*, KIM6 and *Y. enterocolitica* 8081v grown at 37°C. The percentage of PMN cell death following incubation with each strain for the indicated time was determined by comparison to detergent lysed controls. Indicators of cell death measured were release of (**A**) LDH into the supernatant and (**B**) EthD-1 uptake into PMN cells as described in *Materials and Methods*. Results are expressed as the mean ± SEM of three experiments. *, represents difference from PMN controls (*P*<0.05).

To determine if YopJ/YopP was associated with increased cell death in human PMNs, we measured PMN LDH release and membrane integrity following incubation with *Yersinia*. There was no difference (*P*>0.05) in PMN LDH release (Fig. 3a) or EthD-1 staining (Fig. 3b) with any of the strains tested following 3 to 6 h of incubation with *Yersinia*. After 24 h of incubation, KIM6 showed an increase in PMN LDH release and EthD-1 staining compared to the PMN control (Fig. 3a and 3b, e.g., KIM6 induced 24.2±2.4% LDH release and 25.1±8.6% EthD-1 staining compared to PMNs which exhibited 13.2±8.5% LDH release and 12.5±7.3% EthD-1 staining). However, none of the virulence plasmid-containing strains showed an increase in LDH release (Fig. 3a) or EthD-1 staining (Fig. 3b). After 48 h, KIM6 remained the only strain that induced PMN LDH release (61.0±2.9%) or EthD-1 staining (44.0±3.8%) compared to the PMN control (Fig. 3a and 3b).

### Effects of YopJ/YopP on PMN Phosphatidylserine (PS) Exposure

**Figure 4:**
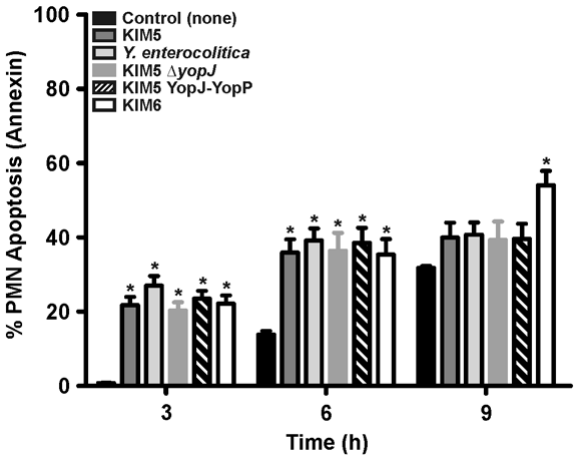
Analysis of PMN phosphatidylserine (PS) externalization. *Y. pestis* KIM5, KIM5 YopJ-YopP, KIM5Δ*yopJ*, KIM6 and *Y. enterocolitica* were grown at 37°C, combined with PMNs, and incubated for the times indicated. PS externalization was determined by annexin V-FITC and flow cytometry following interaction with *Yersinia* Results are expressed as the mean ± SEM of at least three experiments. *, represents difference from PMN controls (*P*<0.05).

To further assess PMN cell viability, we measured externalization of PS to the outer leaflet of the PMN plasma membrane (an early indicator of apoptosis) by flow cytometry analysis of annexin V binding. After 3 h, PMNs incubated with all *Yersinia* strains resulted in ∼25% annexin V positive cells (Fig. 4) and there was no difference (*P*>0.05) in annexin V staining among strains. After 9 h of incubation, the percentage of annexin V positive PMNs incubated with any of the virulence plasmid containing strains did not increase from 6 h; however, there was a ∼22% increase in PMNs stained by annexin V following incubation with KIM6 (54.0±13.4%) compared to the PMN control (31.8±1.6%) or KIM5 (39.0±14.5%) (Fig. 4).

### Neutrophil Caspase Activation in Response to *Yersinia*

**Figure 5:**
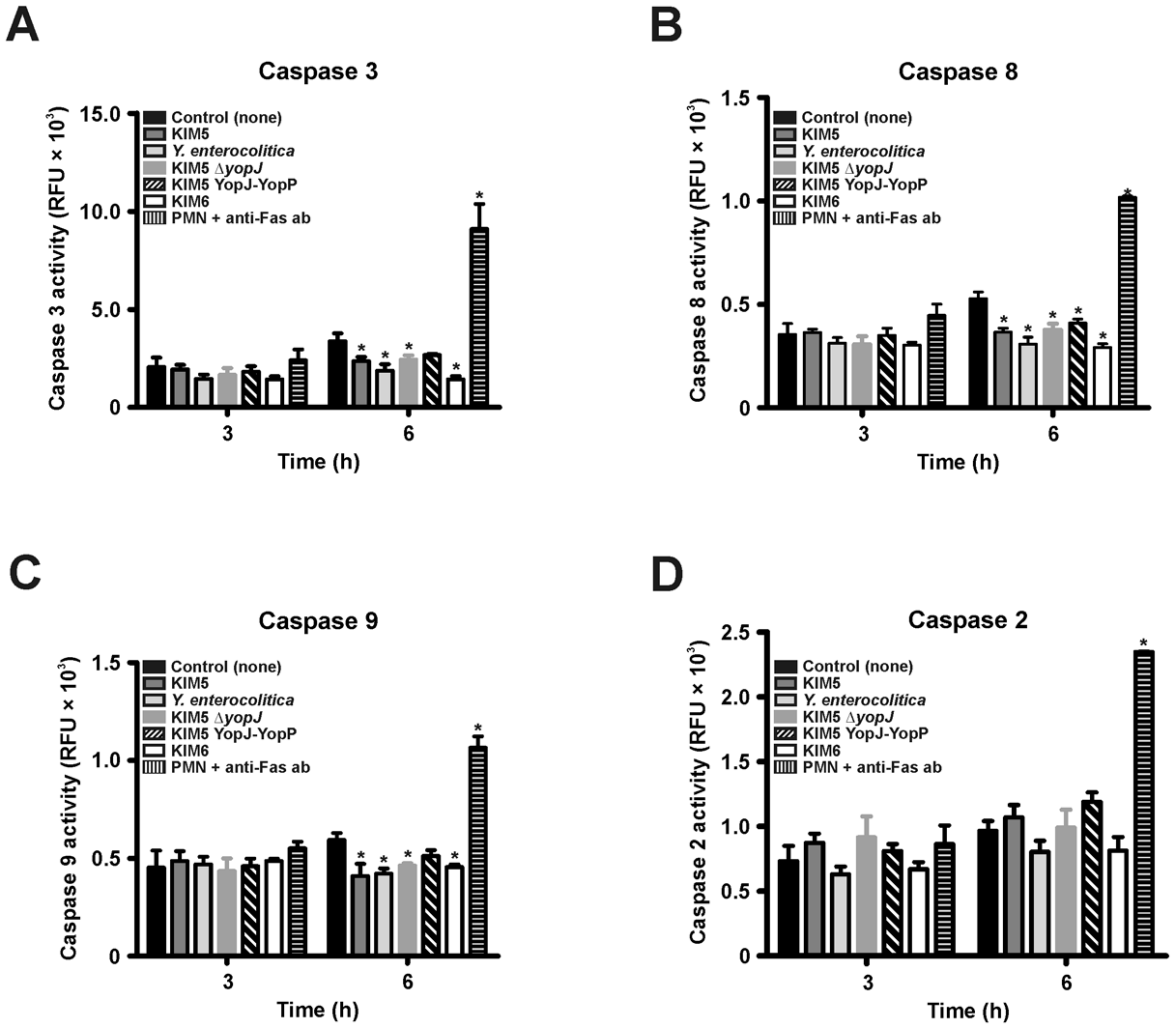
Caspase-3, -8, -9, and -2 activity in PMNs following incubation with *Yersinia*. PMNs were incubated alone or with *Y. pestis* KIM5, KIM5 YopJ-YopP, KIM5Δ*yopJ*, KIM6 and *Y. enterocolitica* 8081v grown at 37°C. Caspase-3, -8, -9, and -2 activity was measured after 3 h and 6 h of incubation as described in *Materials and Methods* and is expressed in relative fluorescence units. Results are expressed as the mean ± SEM of three experiments. *, represents difference from PMN controls (*P*<0.05).

To determine if PMN caspase activation occurs similar to macrophages following interaction with *Yersinia*, we measured PMN caspase-3, -8, -9, and -2 activity after 3 and 6 h of incubation with *Yersinia*. PMNs incubated with anti-Fas antibody were included as a positive control [36]. At 3 h, there was no difference (*P*>0.05) among any strain tested and the PMN control (Fig. 5). At 6 h, there was a 2–3 fold increase in caspase activity due to anti-Fas antibody (Fig. 5). After 6 h, caspase activity in PMNs incubated with any of the *Yersinia* strains was less than or equal to the PMN control (*P*>0.05) (Fig. 5).

### Neutrophil Reactive Oxygen Species (ROS) Production in Response to *Yersinia*

**Figure 6:**
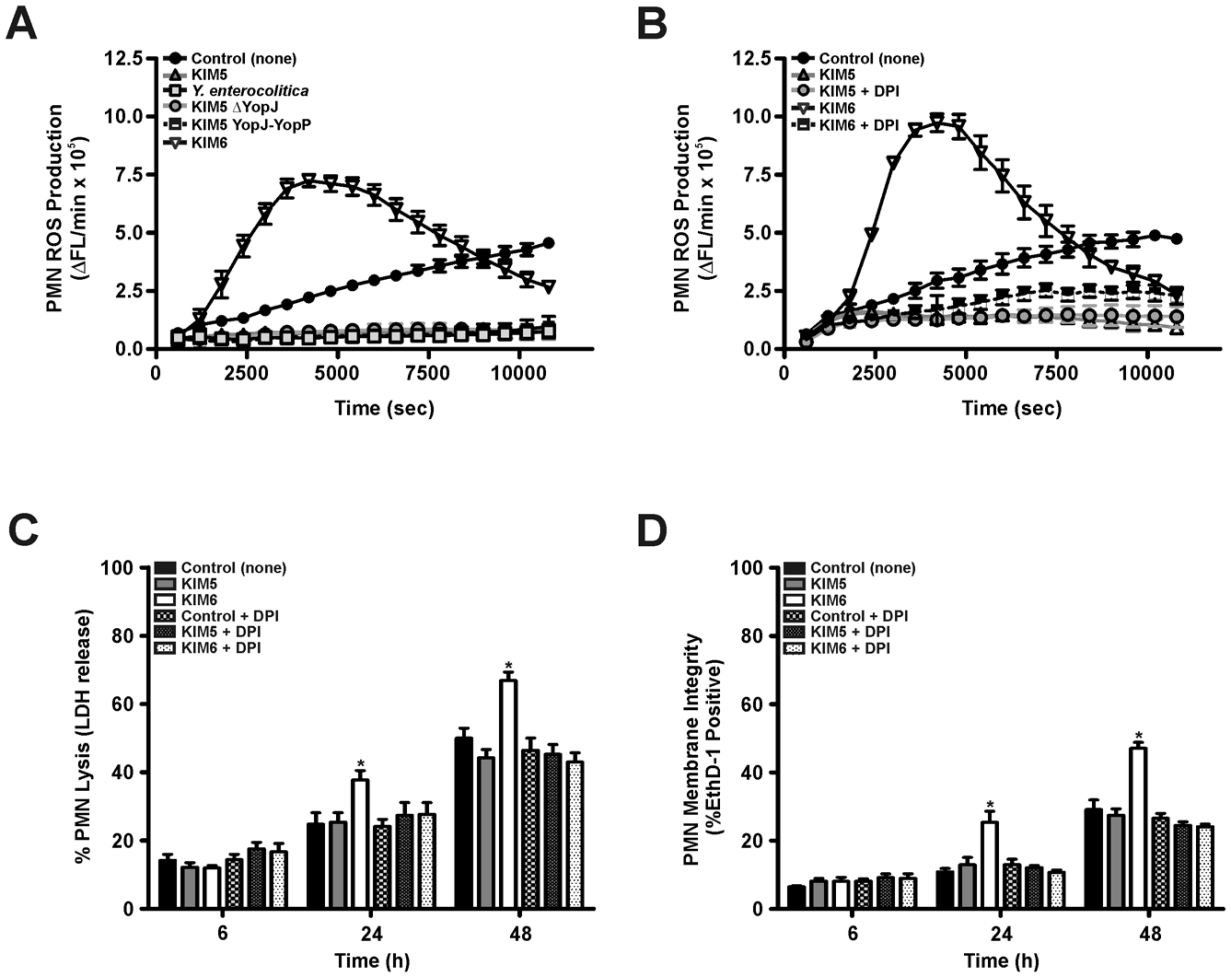
Effects of *Yersinia* on neutrophil ROS production and cell death. PMNs were incubated alone or with *Y. pestis* KIM5, KIM5 YopJ-YopP, KIM5Δ*yopJ*, KIM6 or *Y. enterocolitica* 8081v grown at 37°C. (**A**) PMN ROS production following incubation with the indicated *Yersinia* strains. (**B**) PMN ROS production with or without 10 µM DPI. PMN cell death following phagocytic interaction with *Yersinia* was assessed by (**C**) PMN release of LDH into the supernatant and (**D**) PMN uptake of EthD-1. Results are expressed as the mean ± SEM of three experiments. *, represents difference from PMN controls (*P*<0.05).

PMN externalization of PS and apoptosis are associated with ROS production [6]–[8], [37], [38]. We showed previously that *Y. pestis* strain KIM6 induces rapid PMN ROS production, whereas KIM5 completely inhibits ROS production in human PMNs [32]. To test whether YopJ/YopP play a role in ROS inhibition, PMN ROS was measured following phagocytic interaction with KIM5 YopJ-YopP and KIM5Δ*yopJ*. KIM5 YopJ-YopP and KIM5Δ*yopJ* were found to inhibit PMN ROS production as effectively as KIM5 and *Y. enterocolitica* (Fig. 6a). Since incubation of PMNs with KIM5 resulted in inhibition of PMN ROS production (Fig. 6a) and a decrease in LDH release and EthD-1 staining (Fig. 4), we assessed the contribution of ROS production to PMN cell death. Inhibition of *Y. pestis* KIM6-mediated PMN ROS production with the NADPH oxidase inhibitor DPI was similar to that observed following neutrophil interaction with *Y. pestis* KIM5 (Fig. 6b). In addition, PMNs pretreated with DPI and incubated with KIM6 had decreased levels of LDH release and EthD-1 staining compared to PMN controls incubated with KIM6 (Fig. 6c and 6d, *e.g.*, 42.2±6.6% LDH release and 24.0±1.7% EthD-1 compared to 65.6±6.0% LDH release and 46.8±3.9% EthD-1 staining at 48 h, respectively). Thus, inhibition of ROS production by DPI following 48 h incubation with KIM6 decreased PMN LDH release by ∼23.4% and EthD-1 staining by ∼22.8%. Further, PMN cell death decreased following inhibition of neutrophil ROS production with either DPI or the *Y. pestis* type III secretion system (Fig. 6c and 6d).

## Discussion

The role of *Yersinia* YopJ and YopP in murine macrophage and dendritic cell death is well described [19]–[27]. Notwithstanding, the contribution of *Yersinia* and the role of YopJ and YopP in induction of cell death in human PMNs and HMDMs is less clear. PMNs account for ∼70% of the circulating leukocytes in humans and, along with macrophages, are rapidly recruited to sites of infection where they act to eliminate most invading pathogens [2]. Previous studies show that *Yersinia* expressing YopP are highly cytotoxic to murine macrophages and dendritic cells; however, it was recently demonstrated that amino acid sequence differences between YopJ and YopP result in YopJ being translocated into host cells less efficiently [28], [29]. Consequently, *Yersinia* expressing YopJ are less cytotoxic to J774A.1 cells and dendritic cells than those expressing YopP [28], [29]. Additionally, *Yersinia* engineered to express YopP (instead of YopJ) are less pathogenic to mice via oral [28] or subcutaneous infection [30]. However, *Y. pestis* virulence is maintained following expression of YopP in intravenous and intranasal murine infection models [30] and thus may be involved in transmission. Notwithstanding, these results provide evidence that *Y. pestis* has evolved, at least in part, to limit host innate immune cell apoptosis/cytotoxicity and further demonstrate the importance of understanding host innate immune cell fate following interaction with *Yersinia*.

PMNs are intimately associated with acute inflammation [9], and premature lysis potentiates destruction of host tissue [39]. Based on this premise, at least in part, we investigated the role of *Yersinia* YopJ and YopP in the determination of cell fate in human PMNs. To account for translocation efficiency differences and to analyze YopJ and YopP effects more thoroughly, we created a *Y. pestis* strain expressing YopP in place of YopJ on the native pCD1 virulence plasmid with minimal disruption (KIM5 YopJ-YopP). In macrophages, YopP was the more potent inducer of cell death, although under our assay conditions with synchronized close contact and high bacterial numbers (50∶1 bacteria:PMNs), YopJ also induced caspase activation, EthD-1 staining, and LDH release (Fig. 1 and Fig. 2). Our data also indicate that *Yersinia* induced cell death in HMDMs is similar (albeit delayed and less severe) to that observed for J774A.1 cells (Fig. 1 and Fig. 2). The decreased susceptibility of HMDMs to YopJ/YopP (Fig. 1 and Fig. 2) is in agreement with previous *Y. enterocolitica* findings demonstrating that HMDMs differentiated via colony-stimulating factor, and/or prolonged incubation, exhibit delayed/decreased apoptosis relative to J774A.1 cells [26], [40]. Nonetheless, we found that HMDM and J774A.1 cell death was dependent on YopJ/YopP expression as indicated by severe membrane disruption and release of cytosolic components (Fig. 1 and Fig. 2). By contrast, incubation of *Yersinia* with PMNs yielded drastically different results than those observed with macrophages. Interaction of neutrophils with any of the virulence plasmid carrying *Yersinia* strains did not result in YopJ/YopP induced LDH release, or EthD-1 staining (Fig. 3). However, PMNs incubated with KIM6 lacking a virulence plasmid resulted in increased late PMN LDH release and EthD-1 staining (Fig. 3). Unlike caspase activation observed in macrophages, neither caspase-3, -8, -9, nor -2 activity increased in PMNs incubated with *Yersinia*, indicating that YopJ/YopP did not directly activate the PMN apoptotic pathway (Fig. 5).

PMN apoptosis is associated with increased ROS production [4], [8]. PMNs from chronic granulomatous disease (CGD) patients (NADPH oxidase-deficient) or PMNs treated with DPI (NADPH oxidase inhibitor) exhibit delayed apoptosis in the presence of TNF-α and bacteria [4], [8], [36], [41]. Furthermore, treatment of PMNs with phorbolmyristate acetate (induces robust ROS production), accelerates apoptosis; however, increased levels of ROS inhibit PMN caspase activity [7], [42], [43]. The significance of PMN ROS production following interaction with microbial pathogens *in vivo* was demonstrated in CGD mice in which PMNs fail to externalize PS and undergo apoptosis after infection with pathogens [38]. As a result, PMNs are not cleared from the site of infection, thus increasing *S. aureus* pathogenicity [38]. Previous *Yersinia* studies have shown that KIM5 and *Y. enterocolitica* rapidly and efficiently inhibit PMN ROS production [32], [44], whereas KIM6 stimulates a pronounced ROS response [32]. We found that KIM5 YopJ-YopP and KIM5Δ*yopJ* are able to inhibit neutrophil ROS production as efficiently as KIM5 and *Y. enterocolitica* (Fig. 6a). Inhibition of PMN ROS production by virulence plasmid-containing *Yersinia* strains (Fig. 6a) correlated with the lack of PS exposure (Fig. 4), and subsequent LDH release and EthD-1 staining (Fig. 3) following incubation of *Yersinia* with PMNs. We found that DPI-inhibition of PMN ROS production during phagocytic interaction with *Y. pestis* KIM6 reduced the amount of LDH release and EthD-1 staining (Fig. 6). Thus, our data indicate that ROS contribute to PMN cell death in part, and that *Yersinia* TTSS-dependent inhibition of ROS production delays normal PMN apoptosis.

Overall, our data show that YopJ/YopP-mediated induction of apoptosis does not occur in PMNs in contrast to observations with macrophages. Incubation of KIM5, *Y. enterocolitica*, KIM5 YopJ-YopP, or KIM5Δ*yopJ* with PMNs resulted in decreased PMN exposure of PS, ROS production, LDH release, and EthD-1 staining compared to that of KIM6 (Figs. 3–4, and Fig. 6). On the other hand, PMN interaction with *Y. pestis* strain KIM6 resulted in both apoptosis and cell lysis, which was reduced to KIM5 levels after DPI treatment. These findings suggest that *Yersinia* virulence plasmid-dependent, but YopJ/YopP-independent, inhibition of ROS production alters normal PMN PICD [4], [ 6]–[8]. Pathogen-mediated delay of PMN apoptosis and lysis would plausibly decrease the host inflammatory response, relative to that required for resolution of infection, and facilitate *Yersinia* pathogenesis. This proposed mechanism is consistent with recent findings that demonstrate that increased immune cell cytotoxicity/apoptosis corresponds to attenuation of *Yersinia* virulence [28], [30]. Further studies are required to elucidate *in vivo* the impact of *Y. pestis* on PMN cytokine production, cell fate, and subsequently how altering PICD affects the overall acute inflammatory response during *Yersinia* infection.
